# Multi-omics analysis of hepatic outcomes in T2DM-MAFLD patients treated with semaglutide: a single-centre, longitudinal, data-driven study

**DOI:** 10.3389/fendo.2025.1650729

**Published:** 2025-10-01

**Authors:** Shu Niu, Shuchun Chen, Di Wu, Chenxi Wang, Jianchao Xu, Jiantong Yin, Yubin Zhao

**Affiliations:** ^1^ Department of Endocrine, Shijiazhuang People’s Hospital, Shijiazhuang, Hebei, China; ^2^ Department of Endocrine, Hebei Provincial People’s Hospital, Shijiazhuang, Hebei, China; ^3^ Shijiazhuang Hospital of Traditional Chinese Medicine, Shijiazhuang, Hebei, China; ^4^ Department of Endocrine, Medical University, Shijiazhuang, Hebei, China

**Keywords:** multi-omics analysis, semaglutide, MAFLD-T2DM, hepatic outcomes, proteomics

## Abstract

**Background:**

Metabolic-associated fatty liver disease (MAFLD) is a leading cause of chronic liver disease and is closely linked to type 2 diabetes mellitus (T2DM). The pathogenesis of MAFLD involves complex metabolic imbalances, including impaired fatty acid β-oxidation and chronic inflammation. GLP-1 receptor agonists (GLP-1 RAs) have shown promise in improving metabolic outcomes, but their specific effects on MAFLD remain unclear. This study aims to investigate the molecular mechanisms underlying the hepatic benefits of semaglutide, a GLP-1 RA, in T2DM patients with MAFLD using serum proteomics and metabolomics.

**Methods:**

We conducted a single-centre, longitudinal, data-driven study involving 75 T2DM patients with MAFLD (pre-treatment, PT) and 100 healthy controls (health control, HC). Patients received semaglutide treatment (0.25 mg/week initially, escalated to 0.5 mg/week) for 12 weeks. Serum proteomic and metabolomic profiles were analyzed using 4D-DIA proteomics and LC-MS before and after treatment. Biomarker discovery involved the identification of differential metabolites and proteins, pathway analysis, and integration of proteomic and metabolomic data. Clinical and biochemical parameters were also assessed.

**Results:**

Semaglutide significantly improved metabolic and liver parameters, including HbA1c, BMI, HOMA-IR, liver function, IL-6, and liver stiffness (p < 0.01). Multivariate analysis revealed distinct proteomic and metabolomic profiles between baseline and post-treatment groups. A total of 203 differential metabolites and 61 proteins were identified, with key changes including reductions in long-chain fatty acids and inflammatory mediators, alongside increases in carnitine derivatives and anti-inflammatory proteins. Pathway analysis highlighted effects on fatty acid metabolism, PPAR signaling, and NAFLD-related pathways.

**Conclusion:**

Our study provides a comprehensive multi-omics analysis revealing that semaglutide modulates serum proteomic and metabolomic profiles in T2DM-MAFLD patients, potentially through enhancing mitochondrial β-oxidation, reducing lipid toxicity, and suppressing inflammation. These findings offer mechanistic insights into the hepatic benefits of semaglutide and support its potential as a therapeutic agent for MAFLD. This innovative approach advances our understanding of GLP-1 RAs’ multi-organ protective effects and provides a foundation for developing precision medicine strategies for MAFLD.

## 1 Introduction

Metabolic-associated fatty liver disease (MAFLD), the hepatic manifestation of the metabolic syndrome, has become the leading cause of chronic liver disease worldwide, with a prevalence of up to 20%, and is strongly associated with type 2 diabetes mellitus (T2DM), obesity and insulin resistance (1–3).The hallmarks of MAFLD encompass excessive lipid deposition in hepatocytes, mitochondrial dysfunction, and a persistent inflammatory response. These features can ultimately lead to the development of non-alcoholic steatohepatitis (NASH), liver fibrosis, and even cirrhosis (4–6). These features can ultimately lead to the development of non-alcoholic steatohepatitis (NASH), liver fibrosis, and even cirrhosis.

The pathogenesis of MAFLD is characterised by multiple metabolic imbalances, including enhanced *de novo* synthesis of intrahepatic lipids, impaired fatty acid β-oxidation, and aberrant activation of proinflammatory factors (e.g., IL-6, TGF-β1) (7, 8). Glucagon-like peptide-1 receptor agonists (GLP-1 RAs) represent a novel class of glucagon-like peptide-1 drugs. These agents have been shown to enhance glucose and lipid metabolism by modulating insulin secretion and the appetite centre. Moreover, they have been observed to exert a direct effect on the liver, reducing hepatic steatosis through the inhibition of endoplasmic reticulum stress, the promotion of autophagy, and the restoration of mitochondrial function (9, 10). Of note, the therapeutic benefits of GLP-1 RAs extend beyond glycemic control and weight reduction. Emerging evidence suggests that these agents may exert protective effects across multiple organ systems. For instance, recent studies have indicated that GLP-1 RAs are associated with a lower risk of osteoarthritis and improved joint health, potentially through modulating inflammation and cartilage degradation (11). Furthermore, their anti-inflammatory and immunomodulatory properties may also play a role in reducing the risk or improving outcomes of bone and joint infections (12). These pleiotropic effects underscore the systemic impact of GLP-1 RAs and provide a compelling rationale for investigating their broader protective mechanisms, particularly in the context of multi-system metabolic disorders like MAFLD-T2DM. Of particular note is the long-acting GLP-1 RA semaglutide, which has garnered attention for its substantial weight reduction and cardioprotective properties. However, the precise molecular mechanisms through which it enhances liver outcomes in MAFLD remain to be fully elucidated.

At present, mechanistic studies of type 2 diabetes mellitus (T2DM) in combination with metabolic associated fatty liver disease (MAFLD) are largely confined to single-omics analyses (e.g. metabolome or proteome). This approach is inadequate for the comprehensive revelation of systemic molecular alterations in response to drug intervention (13). Metabolomics has been demonstrated to dynamically reflect the end-phenotypes of genes, proteins and environmental factors acting in combination (14), while proteomics has been shown to resolve the functional regulation of key effector molecules (15). Conducting an integrated analysis of the two can facilitate more precise localisation of disease-related pathways and therapeutic targets. However, there is still a paucity of systematic studies on the effect of semaglutide on the serum multi-omics characteristics of T2DM-MAFLD patients.

The present study utilised 4D-DIA proteomics and LC-MS metabolomics technology to longitudinally analyse the serum molecular characteristics of T2DM patients with MAFLD before and after semaglutide treatment. The objectives of this study are as follows: (1) to reveal the potential biomarkers of semaglutide in improving liver metabolism and inflammation, (2) to clarify the key pathways of its regulation of fatty acid metabolism, mitochondrial function and inflammatory response through multi-omics integration. The provision of a mechanistic basis for the hepatoprotective effect of GLP-1 RAs is of paramount importance, as is the promotion of precise treatment for MAFLD.

## 2 Methods

### 2.1 Research subjects

**Figure 1:**
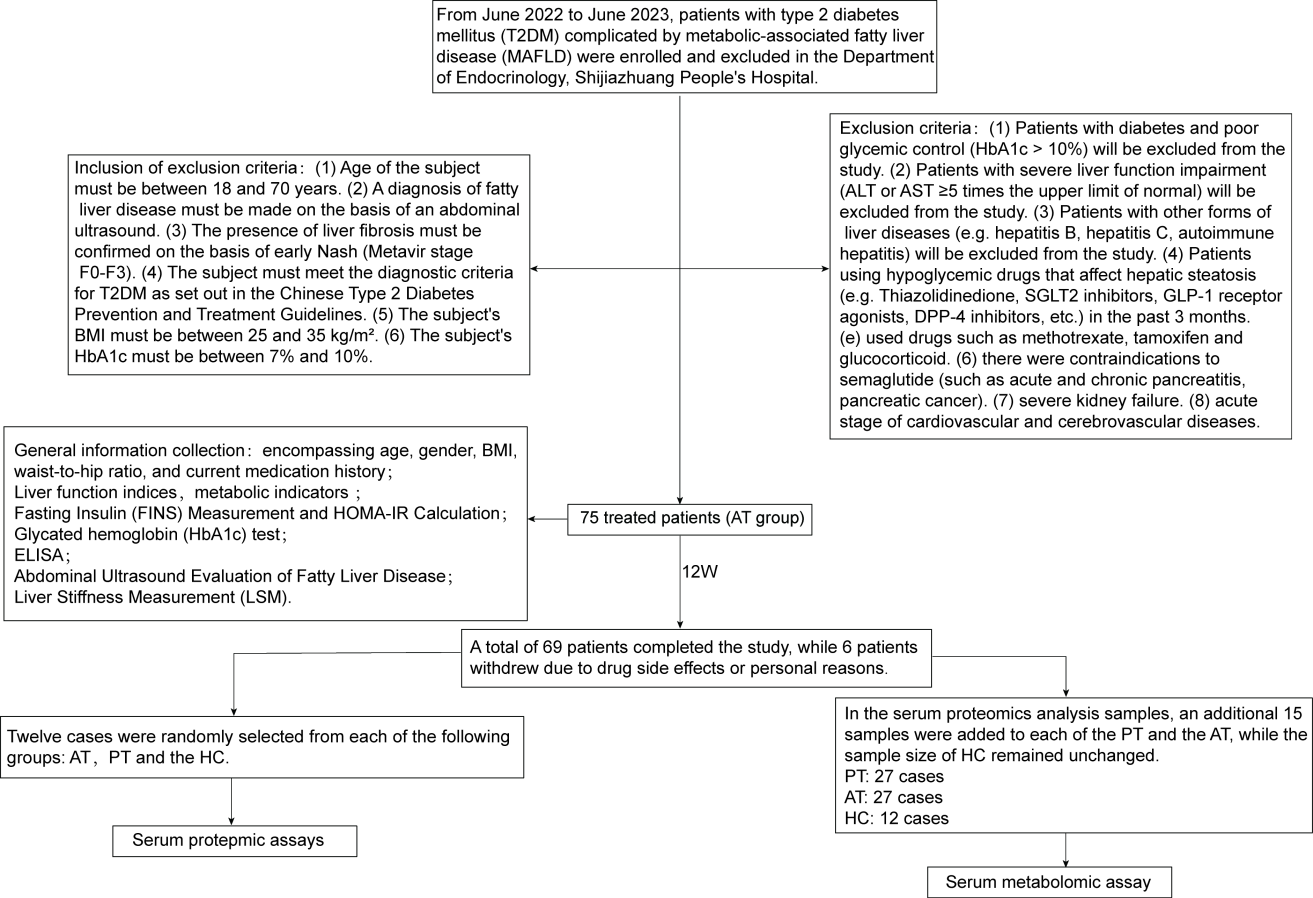
Study flowchart outlining participant recruitment, intervention, and omics analysis. This flowchart outlines the recruitment, inclusion/exclusion criteria, treatment process, and sample selection for proteomic and metabolomic analyses of patients with T2DM-MAFLD from June 2022 to June 2023.

This study was conducted from June 2022 to June 2023 in the Department of Endocrinology at Shijiazhuang People’s Hospital. The final sample comprised 75 patients with T2DM combined with MAFLD who met the specified criteria (PT group), and 100 healthy control subjects (HC group). The study protocol was approved by the Ethics Committee of Shijiazhuang People’s Hospital (Ethics No. [2023]022), and all participants signed an informed consent form. The participant recruitment, grouping, and analysis workflow are summarized in **Figure 1**.

All patients with T2DM combined with MAFLD were treated with subcutaneous injections of semaglutide (Novo Nordisk) in addition to their basal glucose-lowering regimen at an initial dose of 0.25 mg/week, which was increased to 0.5 mg/week after one week. The treatment was continued for 12 weeks. A range of clinical indicators and serum samples were collected from patients before and after treatment.

### 2.2 Inclusion of exclusion criteria

#### 2.2.1 The admission criteria for this study are outlined below

(1) Age of the subject must be between 18 and 70 years. (2) A diagnosis of fatty liver disease must be made on the basis of an abdominal ultrasound. (3) The presence of liver fibrosis must be confirmed on the basis of early Nash (Metavir stage F0-F3). (4) The subject must meet the diagnostic criteria for T2DM as set out in the Chinese Type 2 Diabetes Prevention and Treatment Guidelines. (5) The subject’s BMI must be between 25 and 35 kg/m². (6) The subject’s HbA1c must be between 7% and 10%.

#### 2.2.2 Exclusion criteria

(1) Patients with diabetes and poor glycemic control (HbA1c > 10%) will be excluded from the study. (2) Patients with severe liver function impairment (ALT or AST ≥5 times the upper limit of normal) will be excluded from the study. (3) Patients with other forms of liver diseases (e.g. hepatitis B, hepatitis C, autoimmune hepatitis) will be excluded from the study. (4) Patients using hypoglycemic drugs that affect hepatic steatosis (e.g. Thiazolidinedione, SGLT2 inhibitors, GLP-1 receptor agonists, DPP-4 inhibitors, etc.) in the past 3 months. (e) used drugs such as methotrexate, tamoxifen and glucocorticoid. (6) there were contraindications to semaglutide (such as acute and chronic pancreatitis, pancreatic cancer). (7) severe kidney failure. (8) acute stage of cardiovascular and cerebrovascular diseases.

### 2.3 Collection of clinical data and detection of biochemical indexes

#### 2.3.1 General information collection

A standardised electronic medical record system was utilised to document patients’ baseline information, encompassing age, gender, BMI, waist-to-hip ratio, and current medication history.

The BMI of each participant was calculated as weight (kg)/height² (m²). The electronic weighing scale (accuracy ±0.1 kg) and the wall-mounted height meter (accuracy ±0.5 cm) were utilised for this purpose.

Waist-hip ratio: Waist circumference (the horizontal circumference of the midpoint between the upper edge of the iliac crest and the lower edge of the twelfth rib) and hip circumference (the horizontal circumference of the most prominent point of the buttocks) were measured by using a soft ruler, and the ratio of waist circumference/hip circumference was calculated.

#### 2.3.2 Automatic biochemical analysis and testing

A fully automated biochemical analyser (AU5800, Beckman Coulter, USA) was utilised to ascertain serum biochemical indices, encompassing the following:

Liver function indices: alanine transaminase (ALT), aspartate transaminase (AST), and gamma-glutamyltransferase (GGT).

Furthermore, metabolic indicators such as fasting glucose (FPG), total cholesterol (TC), triglycerides (TG), high-density lipoprotein cholesterol (HDL-C), and low-density lipoprotein cholesterol (LDL-C) were also analysed.

Quality control: Instrument calibration and quality control products (Level 1 & 2, Roche Diagnostics) were performed daily, with an intra-batch coefficient of variation (CV) of less than 5%.

#### 2.3.3 Fasting insulin measurement and HOMA-IR calculation

ASSAY: Serum FINS concentration was quantified by electrochemiluminescence (Cobas e601, Roche Diagnostics, Switzerland) with a sensitivity of 0.2 μIU/mL and a linear range of 1-300 μIU/mL.

HOMA-IR calculation: the steady-state model was evaluated with the formula:

HOMA-IR=FPG (mg/dL)×FINS (μIU/mL)/22.5.

FPG units were converted to mg/dL (1 mmol/L = 18 mg/dL).

#### 2.3.4 Glycated hemoglobin (HbA1c) test

HbA1c was determined by high performance liquid chromatography (HPLC, D-10™, Burroughs, USA) with a detection range of 4.0-18.0% and a batch-to-batch CV of <2%.

#### 2.3.5 ELISA

Cytokine IL-6 (EK1153) was purchased from Hangzhou Lianke Biotechnology Co., Ltd. The absorbance was measured using a full-wavelength enzyme-labeler (Multiskan SkyHigh, Thermo Fisher Scientific, Waltham, MA, USA).

#### 2.3.6 Abdominal ultrasound evaluation of fatty liver disease

The radiologist maintained the confidentiality of other clinical information regarding the participants. Fatty liver was qualitatively evaluated based on the echogenicity of the hepatic parenchyma relative to the right renal cortex. The classification was as follows:

Normal: Homogeneous hepatic echogenicity with no posterior attenuation.

Mild Fatty Liver: Mildly increased hepatic echogenicity compared to the renal cortex, with minimal or no posterior attenuation.

Moderate to Severe Fatty Liver: Markedly increased hepatic echogenicity accompanied by significant posterior attenuation and blurred vascular texture.

#### 2.3.7 Liver stiffness measurement

LSM was performed in the supine position using shear wave elastography (Mindray, Shenzhen, China) through the right intercostal space. Five measurements of liver stiffness were obtained. Liver fibrosis was staged according to the Metavir classification:

F0-F1: LSM < 6 kPa (exclusion of significant fibrosis or cirrhosis).
F2-F3: LSM 6–11.2 kPa (exclusion of cirrhosis but presence of significant fibrosis).
≥11.2 kPa: Cirrhosis.

#### 2.3.8 Follow-up

At the end of the 12-week semaglutide treatment, a total of 69 patients completed the study, while 6 patients withdrew due to drug side effects or personal reasons. The aforementioned indicators were re-measured using the same methods as before.

Subsequently, 12 serum samples were randomly selected from the 69 MAFLD patients who completed the treatment. These samples were designated as the after-treatment (AT) group, representing the serum collected after 12 weeks of treatment. Additionally, 12 serum samples were selected from the 100 patients in the HC group. In total, 36 samples were subjected to serum proteomics analysis.

For metabolomics analysis, 15 additional samples were added to each of the PT and AT groups, while the HC group remained unchanged. This resulted in a total of 66 samples being analyzed for serum metabolomics.

### 2.4 Serum metabolomic assay

#### 2.4.1 Sample preparation and extraction

Samples stored at -80°C were thawed on ice and mixed with the extraction solution containing the internal standard. After vortexing and centrifugation, the supernatant was collected, briefly frozen and then centrifuged a second time. The final supernatant was used for LC-MS analysis, and the volume was adjusted to 180 μL for plasma samples and 120 μL for atrial fluid samples.Metabolite extraction and LC-MS analysis were performed with reference to the International Guidelines for Standardization in Metabolomics (16), using a Waters ACQUITY UPLC BEH C18 column (1.7 μm, 2.1 × 100 mm) with a mobile phase A of 0.1% formic acid aqueous solution, mobile phase B was 0.1% formic acid acetonitrile solution, and the gradient elution program was as follows: 0–2 min 5% B, 2–10 min 5-95% B, 10–12 min 95% B. The separation was performed on a Waters ACQUITY UPLC BEH C18 column (1.7 μm, 2.1×100 mm).

#### 2.4.2 Detection conditions

A liquid chromatography-mass spectrometry (LC-MS) system equipped with a Waters ACQUITY UPLC BEH C18 column was used for the analysis of the samples, with the temperature of the column maintained at 40°C and the flow rate of 0.4 mL/min. The gradient solvent system was started with a water-acetonitrile ratio of 95:5 and gradually varied to different ratios over a period of 10 min. an AB Sciex TripleTOF 6600 mass spectrometer was operated in full MS-scan mode (full MS-scan mode), while data were acquired in positive and negative ionization modes with specific ion source parameters and scanning parameters set, including mass range, integration time, and collision energy. Information-dependent acquisition (IDA) of data was performed using Analyst TF 1.7.1 software.

#### 2.4.3 Metabolic characterization

Raw mass spectrometry data were converted to mzXML format using ProteoWizard software and peak detection and alignment based on mass-to-charge ratio (m/z) and retention time was performed by XCMS software. Metabolites were quantified and calibrated by SVR method. Metabolite identification was accomplished by a four-step method involving internal standard libraries, public databases, and software tools such as MetDNA and CFM-ID. The identification priority was in the following order: standard, MS/MS and MetDNA, and the similarity score threshold for MS/MS spectra matching was set at 0.5.

### 2.5 Serum proteomic assays

#### 2.5.1 Preparation of protein and peptide samples

Samples were thawed on ice, mixed with PBS and PMSF, and subsequently shaken at room temperature to determine total protein concentration using the BCA method. Aliquots of protein samples were subjected to a trypsin digestion process including reduction, alkylation, and precipitation, followed by resuspension and overnight digestion at 37°C. Digested peptides were desalted, dried, and redissolved in formic acid for further analysis.

#### 2.5.2 LC-MS/MS-based proteomics analysis

Liquid chromatography analysis was performed on a nanoElute UHPLC system with a reversed-phase C18 column at 50°C for peptide separation at a flow rate of 0.3 μL/min and a separation time of 60 min. Mobile phase B increased linearly from 2% to 80% within the first 55 min. The liquid chromatography was coupled to a timsTOF Pro2 mass spectrometer running in PASEF mode to acquire MS and MS/MS spectra in the range of 100 to 1700 m/z with a capillary voltage of 1400 V. In diaPASEF mode, the software defined 64 quadrupole isolation windows based on the TIMS scan time and adjusted the collision energies and isolation widths according to the m/z values. Mass spectrometry data acquisition was performed in diaPASEF mode, with collision energies set to 20–50 eV and isolation window widths to 2 Th. Protein identification was performed via the SwissProt database (Release 2023_01), and a reverse database strategy was used to control the false-positive rate (17).

#### 2.5.3 Protein database search

MS raw data were analyzed in a library-free manner using DIA-NN (v1.8.1) software. Spectral libraries were created using the human (Homo sapiens) SwissProt database and deep learning algorithms for neural networks. The MBR option was used to create a spectral library from the DIA data and the data was re-analyzed using this spectral library. The false discovery rate (FDR) of the search results was adjusted to an average of less than 1% for proteins and precursor ionized water, and the remaining identifications were used for subsequent quantitative analysis.

### 2.6 Statistical analysis

This study used SPSS 26.0 statistical analysis software for data analysis. The quantitative data did not obey normal distribution by normality test, and the non-normally distributed variables were expressed as Interquartile range (IQR). Qualitative variables were expressed as percentages. The nonparametric Wilcoxon rank sum test was applied to the nonnormally distributed data, and P<0.05 was considered statistically significant. Differences between metabolic and protein group data were analyzed using the Benjamini-Hochberg method corrected for false discovery rate (FDR < 0.05) (18).

## 3 Result

### 3.1 Clinical and biochemical characteristics

Following a 12-week period of semaglutide treatment, patients diagnosed with T2DM-MAFLD demonstrated substantial enhancements in metabolic and hepatic parameters. Compared to the PT group, the AT group demonstrated significant reductions in HbA1c, BMI, HOMA-IR, liver stiffness (LSM), ALT, AST, γ-GGT, TC, TG, LDL-C, and IL-6 levels (all P-values < 0.01), while HDL-C levels exhibited a significant increase (P-value < 0.05). No significant changes were observed in uric acid (UA) levels (**Tables 1**, **2**). Furthermore, baseline levels of all metabolic and inflammatory markers in the healthy control group (HC group) were found to be significantly lower than those in the PT group (P-values < 0.05) (**Table 1**, **Supplementary Table S1**).

**Table 1:** Clinical characteristics of patients in the normal group (healthy people) and the pre-treatment group (T2DM with MAFLD).

Basic characteristics	ZC-group	ZLQ-group	Z-score	*P*-value
Sample size (n)	100	69		
Age (year)	40.50 (35.00, 45.00)	46.00 (36.00, 54.00)	2.938	2.938
Gender (male,%)	38 (38.0)	49 (71.0)	17.816	<0.001
BMI (kg/m^2^)	21.09 (20.05, 22.78)	28.72 (27.50, 32.07)	-10.768	<0.001
Waist-to-Hip Ratio	0.78 (0.72, 0.86)	0.97 (0.95, 0.99)	-10.939	<0.001
FIB-4	0.83 (0.61, 1.18)	1.11 (0.84, 1.63)	-3.173	0.002
ALT (U/L)	23.30 (17.00, 31.00)	45.00 (40.50, 52.50)	-10.608	<0.001
r-GGT (U/L)	22.50 (16.00, 32.00)	37.00 (28.00, 51.50)	-6.520	<0.001
AST (U/L)	21.50 (17.00, 27.75)	42.00 (39.00, 45.00)	-10.275	<0.001
FPG (mmol/L)	5.09 (4.70, 5.45)	9.60 (7.50, 11.65)	-10.865	<0.001
UA (μmol/L)	281.00 (191.00, 321.00)	378.00 (308.00, 440.00)	7.336	<0.001
TC (mmol/L)	4.18 (3.54, 4.77)	5.94 (5.50, 6.87)	-10.755	<0.001
TG (mmol/L)	1.12(0.88, 1.45)	2.43 (1.94, 3.00)	-10.293	<0.001
HDL-C (mmol/L)	1.41(1.22, 1.52)	1.06 (0.93, 1.17)	-7.772	<0.001
LDL-C (mmol/L)	2.38(1.98, 2.87)	3.91 (3.73, 4.25)	-10.749	<0.001
Bile Acids (μmol/L)	5.20 (3.10, 6.88)	2.80 (1.90, 3.90)	5.479	<0.001
IL-6 (pg/ml)	4.14 (3.97, 4.41)	5.21 (5.00, 5.66)	-10.818	<0.001

**Table 2:** Clinical features of obese T2DM combined with MAFLD before and after treatment.

Basic characteristics	ZC-group	ZLQ-group	Z-score	*P*-value
Sample size(n)	69	69		
BMI (kg/m^2^)	28.72 (27.50, 32.07)	23.65 (22.33, 24.45)	-7.220	<0.001
Waist-to-Hip Ratio	0.97 (0.95, 0.99)	0.85 (0.80, 0.88)	-7.224	<0.001
FIB-4	1.11 (0.84, 1.63)	0.79 (0.57, 1.06)	-5.307	<0.001
ALT (U/L)	45.00(40.50,52.50)	32.00 (23.50, 35.00)	-7.055	<0.001
r-GGT (U/L)	37.00 (28.00, 51.50)	30.00 (22.50, 40.00)	-4.495	<0.001
AST (U/L)	42.00 (39.00, 45.00)	25.00 (21.00, 29.50)	-7.127	<0.001
FPG (mmol/L)	9.60 (7.50, 11.65)	5.70 (5.00, 6.20)	-6.878	<0.001
UA (μmol/L)	378.00 (308.00, 440.00)	367.00 (309.00, 435.00)	0.914	0.361
TC (mmol/L)	5.94 (5.50, 6.87)	4.48 (3.62, 4.92)	-7.059	<0.001
TG (mmol/L)	2.43 (1.94, 3.00)	1.37 (1.02, 1.70)	-6.593	<0.001
HDL-C (mmol/L)	1.06 (0.93, 1.17)	1.42 (1.20, 1.52)	-5.563	<0.001
LDL-C (mmol/L)	3.91 (3.73, 4.25)	2.87 (2.18, 3.10)	-6.994	<0.001
Bile Acids (μmol/L)	2.80 (1.90, 3.90)	2.80 (2.00, 3.85)	7.221	<0.001
IL-6 (pg/ml)	5.21 (5.00, 5.66)	4.17 (3.98, 4.70)	-7.056	<0.001
HbA1c(%)	8.30 (7.15, 9.85)	5.80 (5.15, 6.10)	-6.966	<0.001
FINS(μIU/ml)	19.30 (15.10, 25.90)	7.00 (2.00, 12.95)	-5.977	<0.001
HOMA-IR	9.00 (6.25, 11.80)	5.10 (2.90, 7.95)	-6.987	<0.001
LSM (kPa)	5.78(5.51,6.10)	1.61 (0.91, 2.00)	-7.171	<0.001

### 3.2 Serum metabolomics analysis

**Figure 2:**
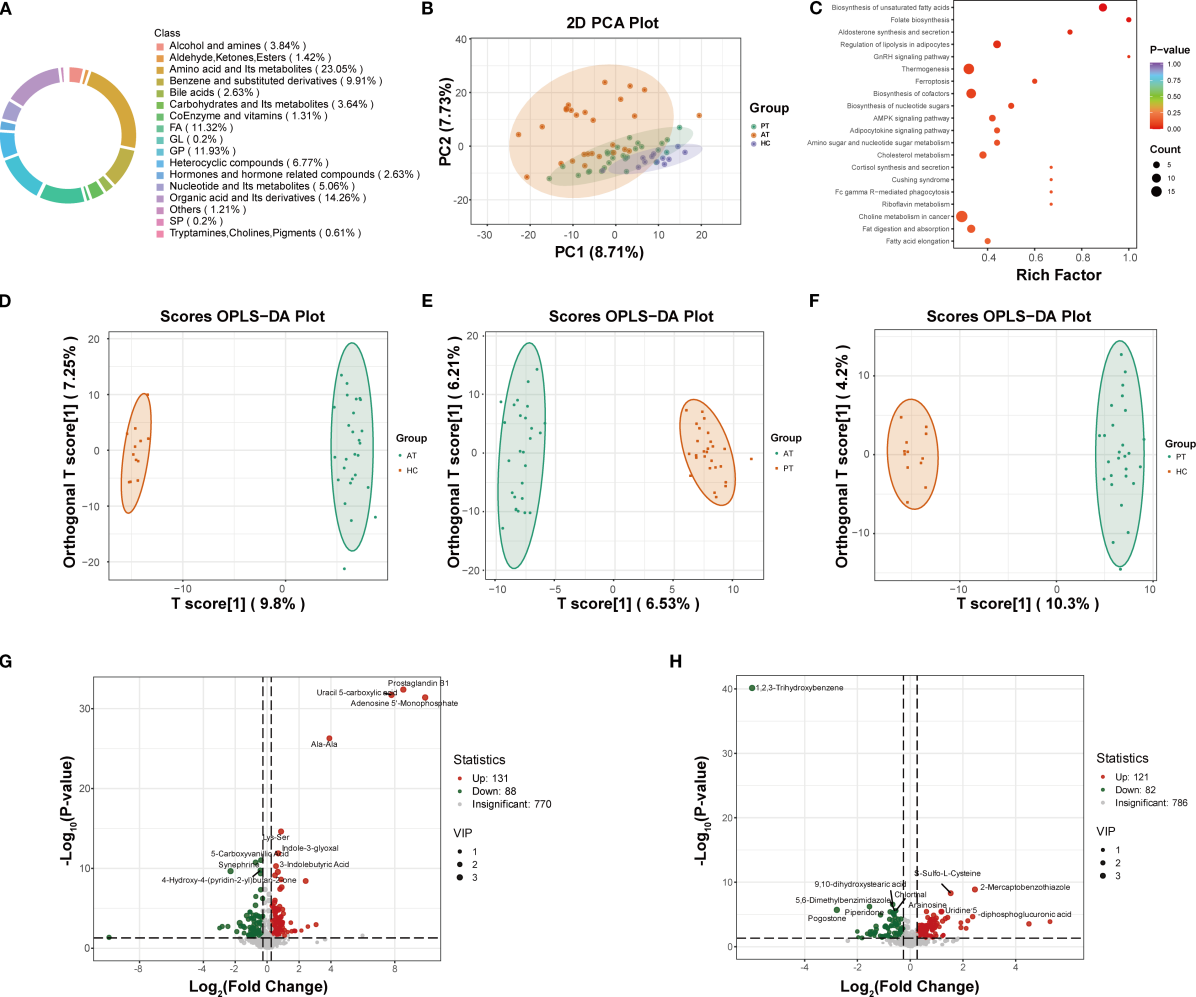
Metabolomic profiling and pathway analysis. **(A)** Circular chart of metabolite category composition (each color represents a metabolite category, and the area of the color block indicates the proportion of that category). **(B)** Principal Component Analysis (PCA) score plot demonstrating distinct clustering of HC, PT, and AT groups. **(C)** KEGG pathway analysis showing enriched pathways related to fatty acid metabolism and NAFLD (P < 0.05). **(D-F)** Orthogonal Partial Least Squares-Discriminant Analysis (OPLS-DA) model validation for AT vs. HC **(D)**, AT vs. PT **(E)**, and PT vs. HC **(F)** groups. **(G)** Volcano plot of differentially expressed metabolites between PT and HC groups. **(H)** Volcano plot of differentially expressed metabolites between AT and PT groups.

Metabolomics analysis revealed significant differences in metabolic profiles among the healthy control group (HC group), baseline MAFLD group (PT group), and post-treatment group (AT group) (**Figure 2A**). Principal component analysis (PCA) demonstrated clear separation between the HC, PT, and AT groups (**Figure 2B**). KEGG pathway analysis indicated that semaglutide modulates pathways related to unsaturated fatty acid biosynthesis, PPAR signaling, and NAFLD (P-value < 0.05) (**Figure 2C**). The R²Y and Q² values of the OPLS-DA model were 0.983 and 0.697, respectively, indicating strong discrimination of metabolic features between groups (**Figures 2D-F**).

#### 3.2.1 Identification and screening of differential metabolites

Differential metabolite analysis identified 219 differentially expressed metabolites (131 of which were found to be expressed at a higher level and 88 at a lower level) between the PT and HC groups (**Figure 2G**), and 203 metabolites (121 of which were found to be expressed at a higher level and 82 at a lower level) between the AT and PT groups (**Figure 2H**). The volcano plot visually summarizes these changes and highlights the top 10 most significantly up- and down-regulated metabolites (**Figures 2G, H**). Compared to the HC group, significantly upregulated metabolites in the PT group included glycerophospholipids (e.g., LPC 0:0/19:0, LPA 0:0/16:0) and pro-inflammatory free fatty acids (e.g., arachidonic acid, prostaglandin B1) (**Table 3**). Following semaglutide treatment (AT group), levels of long-chain fatty acids (e.g., 12,13-EpOME, 9(S)-HODE) were found to be significantly downregulated, while levels of carnitine derivatives (e.g., Carnitine C10:1-OH) were significantly upregulated (**Table 3**). The screening criteria for differential metabolites were set as follows: VIP ≥1.0 and FC ≥1.5 or ≤0.66(**Supplementary Table S2**).

**Table 3:** Differentially expressed metabolites in group comparisons.

Names	Categories	ZC	ZLQ	ZLH	VIP	*P*
12,13-EpOME	FA	32576.99	80191.19	27737.97	1.48	0.01
4-Hydroxy-2-Oxoglutaric Acid	Organic acid and its derivatives	170153.00	228378.70	166567.34	1.89	<0.001
5,6-Dimethylbenzimidazole	Heterocyclic compounds	250420.92	269862.57	181653.51	1.05	<0.001
9(S),12(S),13(S)-TriHOME	FA	35349.17	61130.86	31957.37	2.03	<0.001
9(S)-HODE	FA	71918.91	153250.10	62417.82	2.18	<0.001
DL-3,4-Dihydroxyphenyl glycol	Benzene and substituted derivatives	44482.66	86868.98	47796.93	2.26	<0.001
Dulcitol	Carbohydrates and its metabolites	21044.78	33595.78	20015.07	1.42	0.005
Glycodeoxycholic acid	Bile acids	2806590.77	5186714.38	1890707.41	1.05	0.004
L-Homoarginine	Amino acid and its metabolites	426630.75	564012.96	417440.44	1.02	0.03
Leu-Ala-Val	Amino acid and its metabolites	29109.02	54477.71	35858.90	1.94	0.002
Glycodeoxycholic acid	Amino acid and its metabolites	44537439.88	54702050.79	47012997.48	1.59	0.006
N-Acetyl-L-Tyrosine	Amino acid and its metabolites	167209.15	205909.58	178245.62	1.38	0.004
O-Acetyl-L-homoserine	Amino acid and its metabolites	186699.08	219504.06	181660.00	1.47	0.002
Spermidine	Alcohol and amines	183949.20	229780.98	190279.90	1.38	0.004
Trans-4-Hydroxy-L-Proline	Amino acid and Its metabolites	97458.74	115603.71	91504.37	1.30	0.004
Traumatic acid	Organic acid and its derivatives	275052.52	586723.03	301985.39	1.81	0.02
Tyr-Ala	Amino acid and its metabolites	76804.80	100094.34	65605.66	1.11	0.001
Val-Ala	Amino acid and its metabolites	3341069.35	6461831.30	3326888.57	2.14	<0.001
estrone 3-sulfate	Hormones and hormone-related compounds	178094.55	1045691.35	260407.66	1.54	0.001
2-Amino-3-phosphonopropionic acid	Organic acid and its derivatives	634961.50	478289.74	607289.68	1.23	0.02
2-Aminophenol	Benzene and substituted derivatives	409140.13	285643.66	329695.44	1.70	0.005
2-Hydroxyethanesulfonate	Organic acid and its derivatives	555278.22	414614.00	516418.80	1.69	0.001
4-Hydroxy-3-methoxybenzaldehyde	Benzene and substituted derivatives	840670.99	564811.11	582685.63	1.35	0.0007
4-Hydroxybenzoic Acid	Benzene and substituted derivatives	1239577.51	551946.26	560838.35	1.80	<0.001
4-Hydroxyretinoic Acid	CoEnzyme and vitamins	211542.89	118741.96	147281.11	1.81	0.003
Arg-Phe-Val-Asp	Amino acid and metabolites	19094.23	15177.99	15314.90	1.25	0.003
Corticosterone	Hormones and hormone-related compounds	28645.62	13417.03	16981.50	1.32	0.006
FFA (22:1)	FA	12941458.05	4122480.71	4477635.29	2.32	<0.001
Gly-Phe	Amino acid and its metabolites	2015452.57	1241134.23	1946123.43	1.15	0.01
Guanidinoethyl Sulfonate	Organic acid and its derivatives	138775.35	115191.08	115748.54	1.63	<0.001
Hyp-Thr	Amino acid and its metabolites	414925.03	283973.80	291459.20	2.01	<0.001
Ile-Ala	Amino acid and its metabolites	171623.79	86298.52	100496.58	1.67	<0.001
Ile-Leu	Amino acid and its metabolites	486836.88	301940.82	370448.50	1.28	0.02
Imidazoleacetic acid	Heterocyclic compounds	129760.55	96956.30	125183.57	1.34	0.01
LPC (O-20:3)	GP	398934.09	225390.29	275163.15	2.24	0.01
LPC (O-22:2)	GP	1539101.65	978159.03	1177322.90	2.1	<0.001
LPG (18:0)	GP	100174.38	79241.73	97459.17	1.40	0.008
LPG (20:1)	GP	6744.29	1376.15	1461.64	3.07	<0.001
Oxindole	Heterocyclic compounds	1035187.15	547573.34	891405.68	1.69	0.008
Phe-Gly	Amino acid and its metabolites	1865292.16	1174481.24	1769224.08	1.04	0.02
Piperidine acid	Organic acid and its derivatives	35740774.03	21224954.91	23306468.11	1.75	<0.001
Trp-Glu	Amino acid and its metabolites	48782.13	23819.83	43064.02	1.92	<0.001
Tyrosol	Benzene and substituted derivatives	1444671.09	653398.08	685588.02	1.81	<0.001
Uracil	Nucleotide and its metabolites	987514.82	701753.64	893174.36	1.80	0.0002
Uric acid	Organic acid and its derivatives	41671186.07	23307718.65	38273602.11	1.72	0.0004

#### 3.2.2 Correlation between clinical indices and metabolites

Further analysis was conducted to examine the correlations between HOMA-IR, liver stiffness (LSM), and differential metabolites (**Supplementary Figure S1**). HOMA-IR exhibited positive correlations with metabolites such as 4-Hydroxy-2-Oxoglutaric Acid and 9(S),12(S),13(S)-TriHOME (r > 0, P < 0.05), and negative correlations with metabolites such as 2-Amino-3-phosphonopropionic acid and Gly-Phe (r < 0, P < 0.05) (**Table 4**). LSM showed positive correlations with 12,13-EpOME and traumatin, and a negative correlation with LPC(O-20:3) (**Table 5**). These findings suggest that semaglutide improves insulin resistance and hepatic fibrosis by modulating metabolite levels (**Supplementary Table S3**).

**Table 4:** Correlation analysis between clinical HOMA-IR indicators and metabolites in **Table 3**.

Names	Clinical Indicators	r	*P*
4-Hydroxy-2-Oxoglutaric Acid	HOMA-IR	0.34	0.01
5,6-Dimethylbenzimidazole	HOMA-IR	0.42	0.001
9(S),12(S),13(S)-TriHOME	HOMA-IR	0.37	0.004
DL-3,4-Dihydroxyphenyl glycol	HOMA-IR	0.38	0.003
Dulcitol	HOMA-IR	0.32	0.01
Leu-Ala-Val	HOMA-IR	0.30	0.02
N-Acetyl-L-Tyrosine	HOMA-IR	0.28	0.03
O-Acetyl-L-homoserine	HOMA-IR	0.33	0.01
Tyr-Ala	HOMA-IR	0.32	0.01
Val-Ala	HOMA-IR	0.39	0.003
2-Amino-3-phosphonopropionic acid	HOMA-IR	-0.39	0.003
2-Hydroxyethanesulfonate	HOMA-IR	-0.38	0.004
Gly-Phe	HOMA-IR	-0.41	0.002
Imidazoleacetic acid	HOMA-IR	-0.33	0.01
Phe-Gly	HOMA-IR	-0.37	0.005
Trp-Glu	HOMA-IR	-0.27	0.04
Uric acid	HOMA-IR	-0.50	*P*<0.001

**Table 5:** Correlation analysis between clinical LSM indicators and metabolites in **Table 3**.

Names	Clinical Indicators	r	*P*
12,13-EpOME	LSM	0.37	0.006
4-Hydroxy-2-Oxoglutaric Acid	LSM	0.29	0.03
5,6-Dimethylbenzimidazole	LSM	0.30	0.02
9(S),12(S),13(S)-TriHOME	LSM	0.45	*P*<0.001
9(S)-HODE	LSM	0.57	*P*<0.001
DL-3,4-Dihydroxyphenyl glycol	LSM	0.48	*P*<0.001
Dulcitol	LSM	0.43	0.001
Glycochenodeoxycholic Acid	LSM	0.36	0.006
Lysine	LSM	0.38	0.005
Spermidine	LSM	0.34	0.01
Trans-4-Hydroxy-L-Proline	LSM	0.36	0.006
Traumatic acid	LSM	0.53	*P*<0.001
Val-Ala	LSM	0.50	*P*<0.001
estrone 3-sulfate	LSM	0.32	0.01
2-Amino-3-phosphonopropionic acid	LSM	-0.32	0.01
2-Hydroxyethanesulfonate	LSM	-0.34	0.01
Corticosterone	LSM	-0.30	0.02
FFA(22:1)	LSM	-0.31	0.02
Imidazoleacetic acid	LSM	-0.28	0.03
LPC(O-20:3)	LSM	-0.27	0.04
LPC(O-22:2)	LSM	-0.33	0.01
LPG(18:0)	LSM	-0.31	0.02
Oxindole	LSM	-0.32	0.01

### 3.3 Serum proteomic analysis

**Figure 3:**
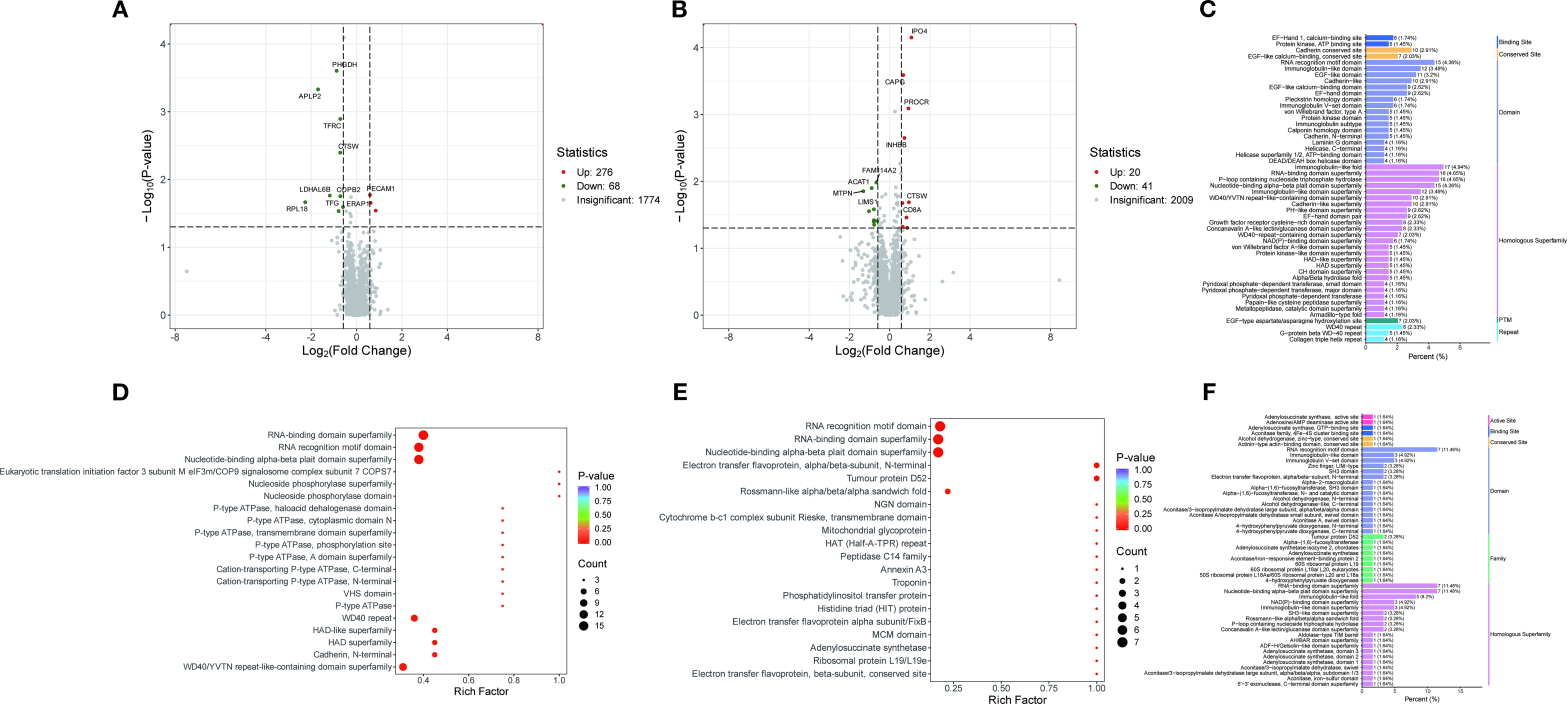
Protein domain and pathway enrichment analysis. **(A)** Volcano plot of differentially expressed proteins between the PT group and the HC group. **(B)** Volcano plot of differentially expressed proteins between the AT group and the PT group. **(C)** Bar plot and bubble plot illustrating protein domain distributions between the PT and HC groups, with enrichment in kinases, peptidases, and phosphatases highlighted. **(D, E)** Bubble plots depicting pathway enrichment for glycolysis/gluconeogenesis **(D)** and mitochondrial β-oxidation **(E)**. **(F)** Protein domain distributions in the comparison between the AT and PT groups.

Proteomic analysis identified 344 differentially expressed proteins (DEPs) between the PT and HC groups, of which 276 were up-regulated and 68 were down-regulated, as well as 61 DEPs between the AT and PT groups, of which 20 were up-regulated and 41 were down-regulated (**Figures 3A, B**; **Supplementary Tables S4** and **S5**).

#### 3.3.1 Differential protein domain analysis

**Figure 4:**
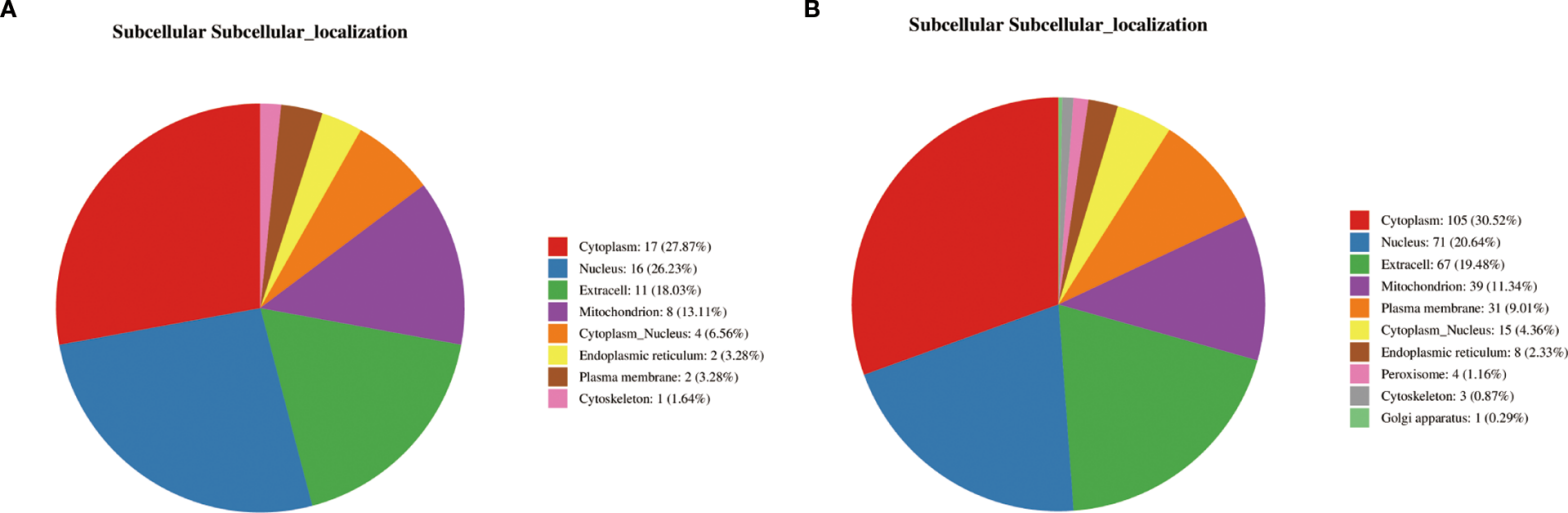
Subcellular localization of differential proteins. **(A)** Subcellular distribution of proteins altered post-semaglutide treatment (AT vs. PT), predominantly localized in the cytoplasm (27.87%) and nucleus (26.23%). **(B)** Localization of differential proteins between PT and HC groups, illustrating compartment-specific metabolic and regulatory functions.

Analysis of the differential protein domains revealed that the prominent domains of the differential proteins between the PT and HC groups were mainly associated with protein kinases, peptidases and phosphatases (**Figure 3C**). These domains are significantly enriched in metabolic pathways such as glycolysis, gluconeogenesis, and Fatty acid metabolism(**Figures 3D, E**). In contrast, the differential proteins between the AT group and the PT group exhibited different domain distributions, with a higher proportion of domains associated with oxidoreductase and chaperones (**Figure 3F**). The detailed distribution of the displayed in **Figure 4**, indicating the distribution of differential proteins in different subcellular structures (**Figure 4**).

#### 3.3.2 GO analysis of differential proteins

**Figure 5:**
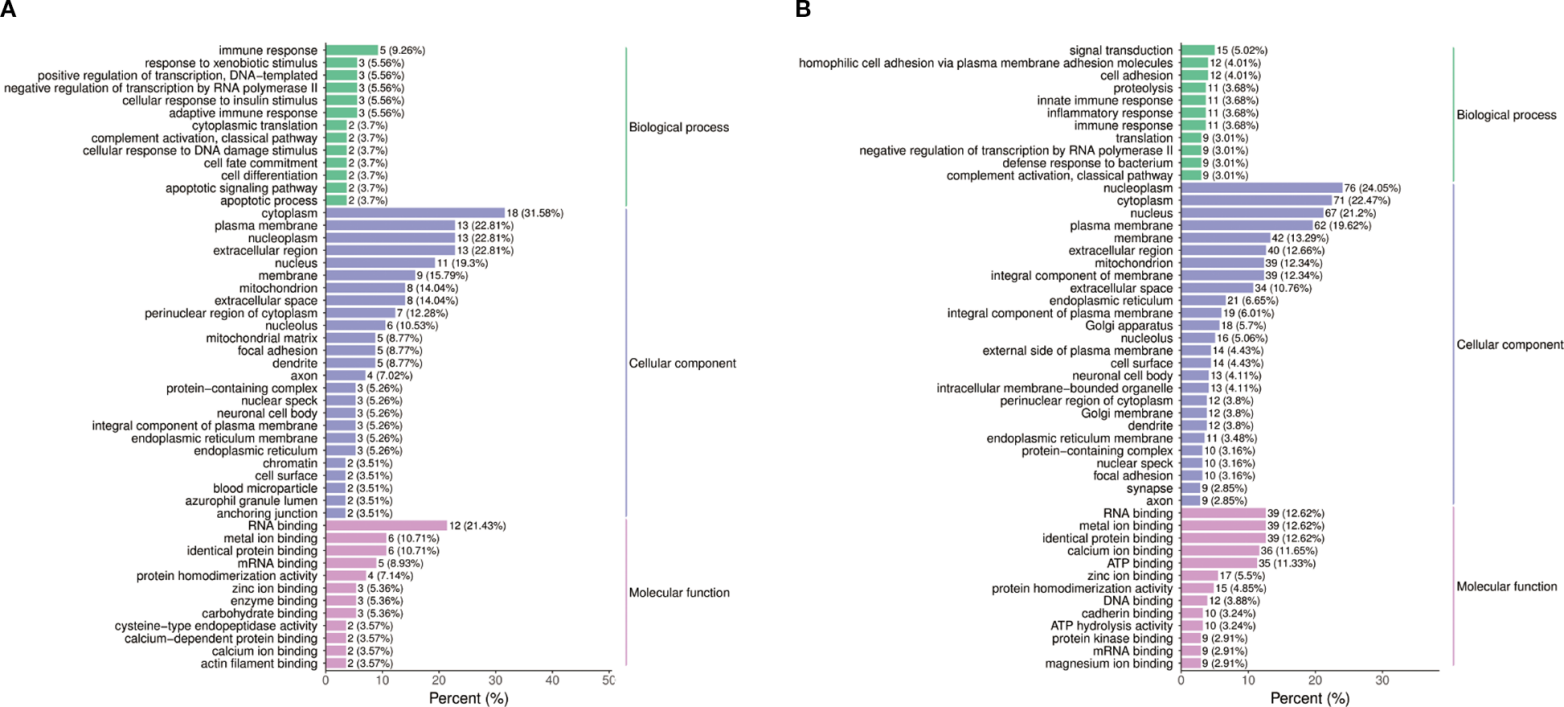
Functional annotation of differential proteins. **(A)** Gene Ontology (GO) analysis of biological processes modulated by semaglutide, including metabolic regulation, cellular organization, and inflammatory response. **(B)** GO classification of proteins differentially expressed in PT vs. HC groups, enriched in lipid metabolism and stress response pathways.

GO analysis of the differential proteins identified several significantly enriched biological processes, including metabolic processes, cellular component organization, and response to stimuli (**Figure 5A**). These findings suggest that the differential proteins identified in this study are associated with a wide range of biological functions and pathways, further revealing the mechanism of the effect of semaglutide on T2DM-MAFLD. The results of Go functional classification analysis of differential proteins in PT and HC groups are displayed in **Figure 5B**, revealing the enrichment of differential proteins in different biological processes.

### 3.4 Proteome-metabolome integration analysis

**Figure 6:**
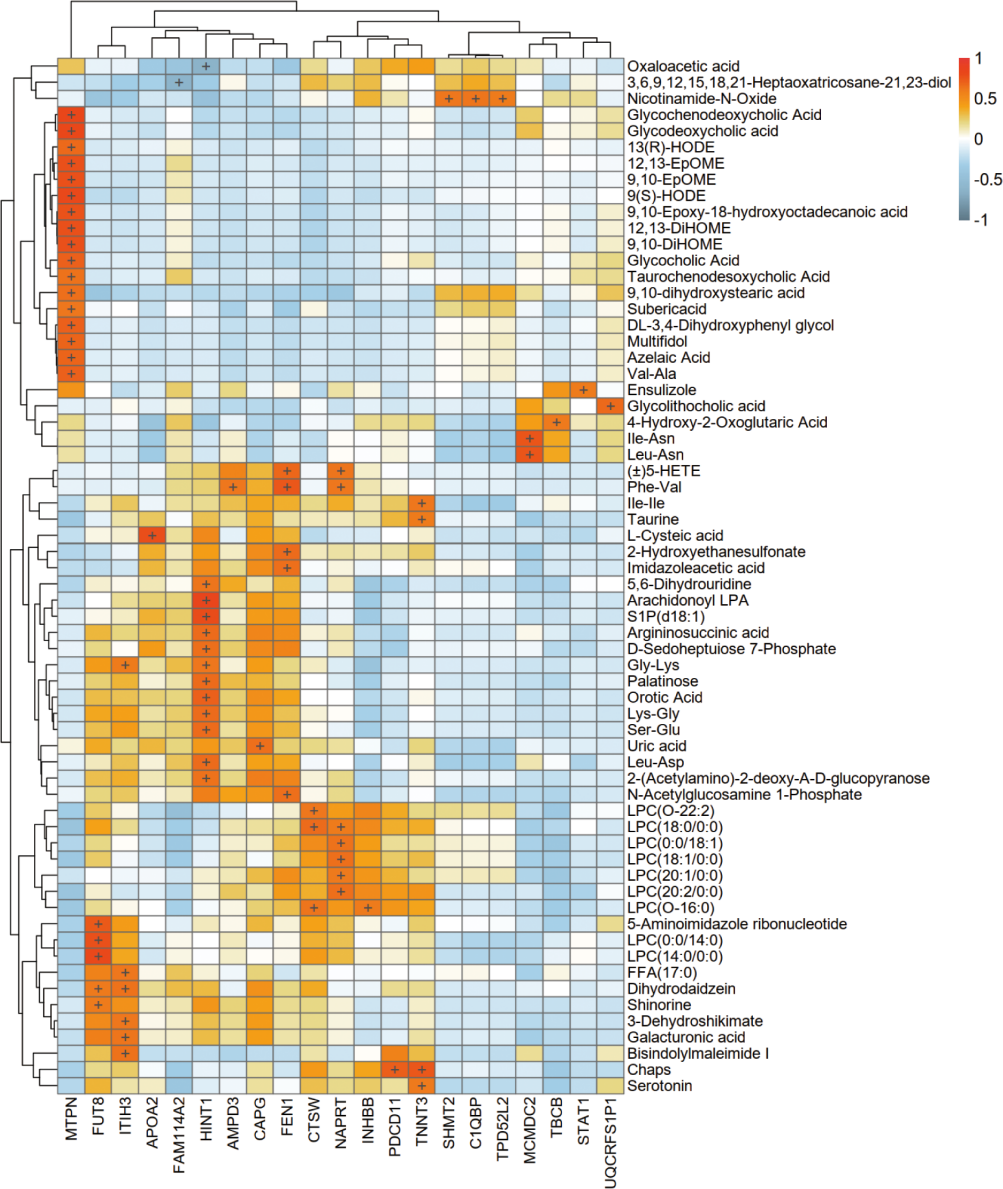
Integrated proteomic-metabolomic network analysis. Heatmap depicting correlations between differentially expressed proteins (horizontal axis) and metabolites (vertical axis) (|r| > 0.6).

Combining proteomic and metabolomic data, it was found that semaglutide affects metabolic pathways by regulating key enzymes and transporters (**Figure 6**). For instance, the upregulation of ITIH3 and INHBB was associated with decreased levels of pro-inflammatory metabolites such as LPC 18:1/0:0. Conversely, the downregulation of ACO1, ACAT1, and STAT1 has been linked to a decrease in lipid synthesis and inflammation. The heatmap in **Figure 5** presents a visual representation of the correlation between differential protein and metabolite expression, with the horizontal axis representing differential proteins, the vertical axis representing differential metabolites, and + representing a correlation with an absolute value greater than 0.6.

## 4 Discussion

In recent years, therapeutic strategies for metabolic dysfunction-associated fatty liver disease (MAFLD) have evolved from single-target interventions to multi-target synergistic regulation, addressing its complex metabolic-inflammatory-fibrotic pathological network. This study demonstrates that obese T2DM patients with MAFLD exhibit significant glucolipid metabolic disorders, impaired islet function, hepatic dysfunction, and elevated inflammatory markers, including BMI, FPG, FINS, HOMA-IR, HbA1c, TC, TG, LDL-C, ALT, AST, and IL-6 levels, all significantly higher than healthy controls. Semaglutide intervention substantially improved these parameters. Multivariate analysis models further revealed distinct separations in metabolomic and proteomic profiles among healthy controls, baseline MAFLD patients, and post-treatment groups, indicating that semaglutide-induced systemic molecular remodeling constitutes a key mechanism for hepatic outcome improvement.

Metabolomic profiling identified significant upregulation of glycerophospholipids (e.g., LPC 0:0/19:0, LPA 0:0/16:0) and pro-inflammatory free fatty acids (FFAs, including arachidonic acid and prostaglandin B1) in MAFLD patients. Glycerophospholipids contribute to MAFLD progression through oxidative stress activation and pro-inflammatory cytokine induction (e.g., IL-6, TNF-α) (19), while excessive FFAs suppress mitochondrial β-oxidation and induce hepatocyte lipotoxicity (20). Semaglutide treatment significantly reduced long-chain fatty acids (12,13-EpOME, 9(S)-HODE) while enhancing carnitine derivatives (Carnitine C10:1-OH). Carnitine facilitates mitochondrial fatty acid transport to enhance β-oxidation capacity and reduce lipotoxic intermediate accumulation (e.g., ceramides) (21), aligning with GLP-1 RAs’ mechanism of AMPK-PPARα pathway activation for lipid synthesis/decomposition balance (9), suggesting semaglutide’s “dual regulatory” effects on hepatic lipid metabolism.

Proteomic analysis revealed semaglutide-mediated downregulation of inflammatory mediators (STAT1, TGFB1I1) and apoptotic executor CASP3. STAT1, a core regulator of interferon signaling, drives MAFLD-to-NASH progression (22), while CASP3 promotes hepatocyte apoptosis and fibroinflammatory cascades under lipotoxic stress (23). The observed CASP3 reduction correlated with IL-6 attenuation and liver stiffness improvement, suggesting semaglutide interrupts the “metabolic-inflammatory-fibrotic” vicious cycle. Notably, semaglutide reversed MAFLD-associated SRSF1 upregulation, a splicing factor exacerbating FFA-induced hepatocyte inflammation (24).

Semaglutide elevated branched-chain amino acids (BCAAs: valine, leucine, isoleucine), potentially inhibiting lipogenic genes (e.g., SREBP-1c) via AMPK activation while enhancing β-oxidation (25). Concurrent increases in serine and taurine may mitigate oxidative stress through glutathione system restoration and SOD activity enhancement (26, 27). Intriguingly, semaglutide upregulated ITIH3 expression, a mitochondrial respiration enhancer inversely associated with MAFLD severity (28), and downregulated ACAT1, suggesting improved metabolic homeostasis through ketogenesis-lipolysis balance modulation (29).

Integrated multi-omics analysis demonstrated semaglutide’s predominant effects on fatty acid metabolism and NAFLD-related pathways. ITIH3 upregulation positively correlated with anti-inflammatory metabolites (Bisindolylmaleimide I) and antioxidant polysaccharides (galacturonic acid), indicating potential downstream hepatoprotective mechanisms. INHBB upregulation, negatively associated with Gly-Lys levels, may ameliorate metabolic dysregulation through anti-inflammatory cytokine modulation.

This study has several limitations. First, although we identified significant alterations in pathways such as fatty acid β-oxidation and PPAR signaling, we lacked direct functional validation through experiments such as mitochondrial respiration assays, gene expression analysis of key regulators (e.g., PPARα target genes), or isotopic tracing of fatty acid oxidation. Second, as a single-center study with limited omics sample size, future validation in larger cohorts is warranted to confirm ITIH3 and ACAT1 as biomarkers. Mechanistic studies using ITIH3 knockout models and investigations into semaglutide-SGLT2 inhibitor combination therapies hold significant clinical implications.

## 5 Conclusion

In summary, the present study analyzed the molecular network of semaglutide to improve the hepatic outcome of T2DM-MAFLD at a multi-omics level, and revealed the innovative mechanism of its therapeutic effects through the metabolic-inflammatory-mitochondrial axis. These findings not only deepen the understanding of the multi-organ protective effects of GLP-1 RAs, but also provide a theoretical basis for the development of MAFLD therapeutic strategies based on multi-target modulation. Future studies could further explore the combination of semaglutide with other metabolic modulators (e.g., SGLT2 inhibitors) with the aim of achieving better clinical outcomes.

## Data Availability

The original contributions presented in the study are included in the article/**Supplementary Material**. Further inquiries can be directed to the corresponding author.

## Supplementary material

Supplementary Figure 1 Correlation analysis among clinical indicators, specific metabolites, and proteins. **(A)** PT group vs. HC group. **(B)** AT group vs. HC group.

Supplementary Table 1 Demographic and clinical characteristics of study participants.

Supplementary Table 2 Differentially expressed metabolites between groups.

Supplementary Table 3 Correlation between metabolites and clinical indicators.

Supplementary Table 4 Differentially expressed proteins between PT and HC groups.

Supplementary Table 5 GO enrichment analysis of differentially expressed proteins.

## References

[1] Eslam M Sanyal AJ George J. MAFLD: A consensus-driven proposed nomenclature for metabolic associated fatty liver disease. Gastroenterology. (2020) 158:1999–2014.e1. doi: 10.1053/j.gastro.2019.11.312, PMID: 32044314

[2] Younossi ZM Koenig AB Abdelatif D Fazel Y Henry L Wymer M. Global epidemiology of nonalcoholic fatty liver disease-Meta-analytic assessment of prevalence, incidence, and outcomes. Hepatology. (2016) 64:73–84. doi: 10.1002/hep.28431, PMID: 26707365

[3] Mantovani A Byrne CD Bonora E Targher G. Nonalcoholic fatty liver disease and risk of incident type 2 diabetes: A meta-analysis. Diabetes Care. (2018) 41:372–82. doi: 10.2337/dc17-1902, PMID: 29358469

[4] Friedman SL Neuschwander-Tetri BA Rinella M Sanyal AJ. Mechanisms of NAFLD development and therapeutic strategies. Nat Med. (2018) 24:908–22. doi: 10.1038/s41591-018-0104-9, PMID: 29967350 PMC6553468

[5] Targher G Corey KE Byrne CD Roden M. The complex link between NAFLD and type 2 diabetes mellitus - mechanisms and treatments. Nat Rev Gastroenterol Hepatol. (2021) 18:599–612. doi: 10.1038/s41575-021-00448-y, PMID: 33972770

[6] Adams LA Anstee QM Tilg H Targher G. Non-alcoholic fatty liver disease and its relationship with cardiovascular disease and other extrahepatic diseases. Gut. (2017) 66:1138–53. doi: 10.1136/gutjnl-2017-313884, PMID: 28314735

[7] Softic S Cohen DE Kahn CR. Role of dietary fructose and hepatic *de novo* lipogenesis in fatty liver disease. Dig Dis Sci. (2016) 61:1282–93. doi: 10.1007/s10620-016-4054-0, PMID: 26856717 PMC4838515

[8] Tilg H Moschen AR Roden M. NAFLD and diabetes mellitus. Nat Rev Gastroenterol Hepatol. (2017) 14:32–42. doi: 10.1038/nrgastro.2016.147, PMID: 27729660

[9] Armstrong MJ Gaunt P Aithal GP Barton D Hull D Parker R. Liraglutide safety and efficacy in patients with non-alcoholic steatohepatitis (LEAN): a multicentre, double-blind, randomised, placebo-controlled phase 2 study. Lancet. (2016) 387:679–90. doi: 10.1016/S0140-6736(15)00803-X, PMID: 26608256

[10] Smits MM Muskiet MH Tonneijck L Kramer MH Diamant M van Raalte DH. GLP-1 receptor agonist exenatide increases capillary perfusion independent of nitric oxide in healthy overweight men. Arterioscler Thromb Vasc Biol. (2015) 35:1538–43. doi: 10.1161/ATVBAHA.115.305447, PMID: 25908765

[11] Ryan M Megyeri S Nuffer W Trujillo JM. The potential role of GLP-1 receptor agonists in osteoarthritis. Pharmacotherapy. (2025) 45:177–86. doi: 10.1002/phar.70005, PMID: 39980227

[12] Kim BI LaValva SM Parks ML Sculco PK Della Valle AG Lee GC. Glucagon-like peptide-1 receptor agonists decrease medical and surgical complications in morbidly obese patients undergoing primary TKA. J Bone Joint Surg Am. (2025) 107:348–55. doi: 10.2106/JBJS.24.00468, PMID: 39719003

[13] Goyal NP Schwimmer JB. The genetics of pediatric nonalcoholic fatty liver disease. Clin Liver Dis. (2018) 22:59–71. doi: 10.1016/j.cld.2017.08.002, PMID: 29128061 PMC5693307

[14] Johnson CH Ivanisevic J Siuzdak G. Metabolomics: beyond biomarkers and towards mechanisms. Nat Rev Mol Cell Biol. (2016) 17:451–9. doi: 10.1038/nrm.2016.25, PMID: 26979502 PMC5729912

[15] Aebersold R Mann M. Mass-spectrometric exploration of proteome structure and function. Nature. (2016) 537:347–55. doi: 10.1038/nature19949, PMID: 27629641

[16] Sumner LW Amberg A Barrett D Beale MH Beger R Daykin CA. Proposed minimum reporting standards for chemical analysis Chemical Analysis Working Group (CAWG) Metabolomics Standards Initiative (MSI). Metabolomics. (2007) 3:211–21. doi: 10.1007/s11306-007-0082-2, PMID: 24039616 PMC3772505

[17] Elias JE Gygi SP. Target-decoy search strategy for increased confidence in large-scale protein identifications by mass spectrometry. Nat Methods. (2007) 4:207–14. doi: 10.1038/nmeth1019, PMID: 17327847

[18] Benjamini Y Hochberg Y. Controlling the false discovery rate: A practical and powerful approach to multiple testing. J R Stat Soc: Ser B (Methodol). (2018) 57:289–300. doi: 10.1111/j.2517-6161.1995.tb02031.x

[19] Puri P Wiest MM Cheung O Mirshahi F Sargeant C Min HK. The plasma lipidomic signature of nonalcoholic steatohepatitis. Hepatology. (2009) 50:1827–38. doi: 10.1002/hep.23229, PMID: 19937697 PMC5031239

[20] Cusi K Sanyal AJ Zhang S Hartman ML Bue-Valleskey JM Hoogwerf BJ. Non-alcoholic fatty liver disease (NAFLD) prevalence and its metabolic associations in patients with type 1 diabetes and type 2 diabetes. Diabetes Obes Metab. (2017) 19:1630–4. doi: 10.1111/dom.12973, PMID: 28417532

[21] Reuter SE Evans AM. Carnitine and acylcarnitines: pharmacokinetic, pharmacological and clinical aspects. Clin Pharmacokinet. (2012) 51:553–72. doi: 10.1007/BF03261931, PMID: 22804748

[22] Stienstra R Mandard S Tan NS Wahli W Trautwein C Richardson TA. The Interleukin-1 receptor antagonist is a direct target gene of PPARalpha in liver. J Hepatol. (2007) 46:869–77. doi: 10.1016/j.jhep.2006.11.019, PMID: 17321000

[23] Schuster S Cabrera D Arrese M Feldstein AE. Triggering and resolution of inflammation in NASH. Nat Rev Gastroenterol Hepatol. (2018) 15:349–64. doi: 10.1038/s41575-018-0009-6, PMID: 29740166

[24] Yuan N Shen L Peng Q Sha R Wang Z Xie Z. SRSF1 is required for mitochondrial homeostasis and thermogenic functi on in brown adipocytes through its control of ndufs3 splicing. Adv Sci. 11. doi: 10.1002/advs.202306871, PMID: 38569495 PMC11151030

[25] Newgard CB An J Bain JR Muehlbauer MJ Stevens RD Lien LF. A branched-chain amino acid-related metabolic signature that differentiates obese and lean humans and contributes to insulin resistance. Cell Metab. (2009) 9:311–26. doi: 10.1016/j.cmet.2009.02.002, PMID: 19356713 PMC3640280

[26] Sun M Gu Y Glisan SL Lambert JD. Dietary cocoa ameliorates non-alcoholic fatty liver disease and increa ses markers of antioxidant response and mitochondrial biogenesis in hi gh fat-fed mice. J Nutr Biochem. (2021) 92:108618. doi: 10.1016/j.jnutbio.2021.108618, PMID: 33711421 PMC13185143

[27] Matsui M Tanaka K Higashiguchi N Okawa H Yamada Y Tanaka K. Protective and therapeutic effects of fucoxanthin against sunburn caus ed by UV irradiation. J Pharmacol Sci. (2016) 132:55–64. doi: 10.1016/j.jnutbio.2021.108618, PMID: 27590588

[28] Talari NK Mattam U Kaminska D Sotomayor-Rodriguez I Rahman AP Péterfy M. Hepatokine ITIH3 protects against hepatic steatosis by downregulating mitochondrial bioenergetics and *de novo* lipogenesis. iScience. (2024) 27:109709. doi: 10.1016/j.isci.2024.109709, PMID: 38689636 PMC11059128

[29] Houten SM Violante S Ventura FV Wanders RJ. The biochemistry and physiology of mitochondrial fatty acid β-oxidation and its genetic disorders. Annu Rev Physiol. (2016) 78:23–44. doi: 10.1146/annurev-physiol-021115-105045, PMID: 26474213

