# Supplementary figures and images for: Multi-omics analysis of hepatic outcomes in T2DM-MAFLD patients treated with semaglutide: a single-centre, longitudinal, data-driven study
Source: Front Endocrinol (Lausanne). 2025 Oct 1;16:1650729. doi: 10.3389/fendo.2025.1650729 (PMC12521249; doi:10.3389/fendo.2025.1650729)

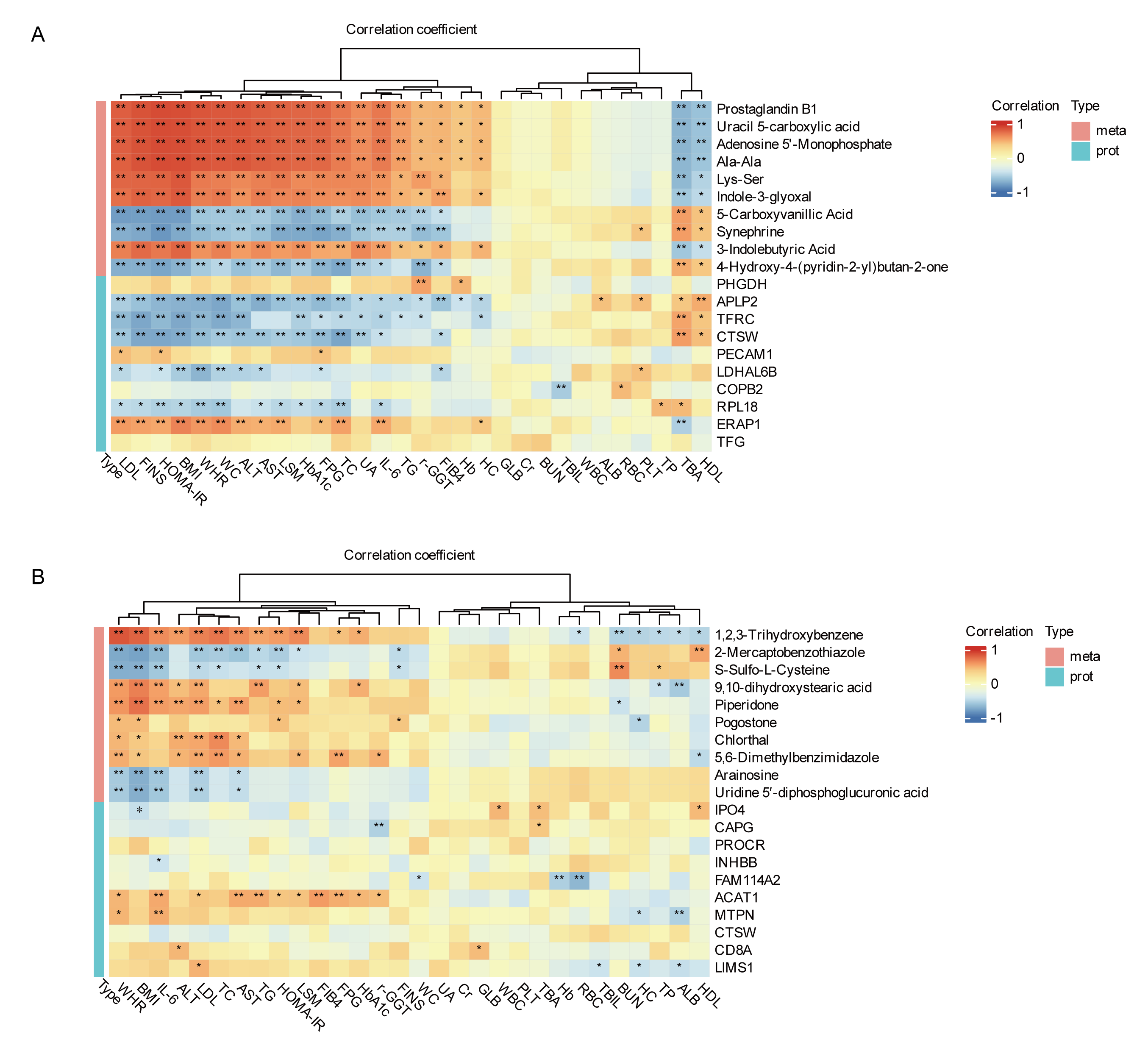

Supplement: Supplementary Figure 1 — Correlation analysis among clinical indicators, specific metabolites, and proteins. (A) PT group vs. HC group. (B) AT group vs. HC group. [file Image1.tif]
